# RNAi-Induced Gene Silencing against Chikungunya and COVID-19: What Have We Learned So Far, and What Is the Way Forward?

**DOI:** 10.3390/v16091489

**Published:** 2024-09-20

**Authors:** Kingshuk Panda, Kalichamy Alagarasu, Rajarshee Tagore, Mandar Paingankar, Satyendra Kumar, Manish Kumar Jeengar, Sarah Cherian, Deepti Parashar

**Affiliations:** 1 Dengue & Chikungunya Group, ICMR-National Institute of Virology, 20-A, Dr. Ambedkar Road, Pune 411001, India; kingshukpanda7@gmail.com (K.P.); alagarasu@gmail.com (K.A.); rajarsheetagore@gmail.com (R.T.); mandarpaingankar@gmail.com (M.P.); prashersk@gmail.com (S.K.); manish.pharmacology@live.in (M.K.J.); 2 Academy of Scientific and Innovative Research (AcSIR), Ghaziabad 201002, India; sarahcherian100@gmail.com; 3 Bioinformatics Group, ICMR-National Institute of Virology, 20-A, Dr. Ambedkar Road, Pune 411001, India

**Keywords:** RNA interference, siRNA, Chikungunya, SARS-CoV-2

## Abstract

RNA interference (RNAi) is a process in which small RNA molecules (such as small interfering RNAs or siRNAs) bind to specific messenger RNAs (mRNAs), leading to its degradation and inhibition of protein synthesis. Our studies have shown that RNAi can effectively silence genes involved in the replication of the Chikungunya virus (CHIKV) in cells. However, these investigations were performed only in laboratory settings and have yet to be tested in human clinical trials. Researchers need to conduct more research to determine the safety and efficacy of RNAi-based therapies as a therapeutic agent to treat viral infections. In this review, the history of evolution of siRNA as an inhibitor of protein synthesis, along with its current developments, is discussed based on our experience. Moreover, this review examines the hurdles and future implications associated with siRNA based therapeutic approaches.

## 1. Introduction

In 1978, Zamecnik first reported that a synthetic oligonucleotide complementary to Rous sarcoma virus 35_S_ RNA acted as an efficient inhibitor of protein expression [1]. At that time, the use of oligonucleotides to inhibit gene expression was termed as antisense technology. In 1998, Fire et al. reported that a double-stranded RNA could potentially and specifically interfere with the expression of an endogenous gene at the mRNA level, leading to its silencing in *Caenorhabditis elegans* [2]. After three years, Elbashir et al. reported that a 21-22nt dsRNA could cause gene silencing without eliciting any non-specific immune response in *Drosophila melanogaster* embryo lysate [3]. Later, it was shown that mutant flies, which were defective in the dsRNA uptake pathway, were susceptible to infection with viruses, suggesting the importance of RNAi in antiviral immunity in insects [4]. The antiviral role of RNAi mechanisms in mammals has been debated in the past [5]. However, the role of antiviral RNAi become clear when it was shown that the genetic ablation of viral suppressors of RNAi reduced Nodamura virus accumulation, but not in RNAi-deficient mouse cells [6,7]. The finding that mouse embryonic stem cells produced increased amounts of siRNA compared to differentiated cells also suggested that somatic cells rely on the interferon response while undifferentiated stem cells use RNAi pathways for antiviral immunity [6,7].

Even before the clear-cut establishment of the antiviral role of the RNAi mechanism in mammals, many programs explored the use of RNAi to treat certain diseases. RNAi modalities, e.g., small interfering RNA (siRNA) and microRNA (miRNA), can knock down the expression of a target gene through mRNA degradation in a sequence-specific way. [8]. Additionally, miRNA can knock down the expression of a particular gene by repression of translated mRNA [9]. During the early stage of developing siRNA therapeutics, researchers designed an unmodified compact siRNA that could bind a target region [10]. Later on, it was observed that unmodified siRNA had several disadvantages: susceptibility to ribonuclease digestion, short biological half-life, lack of proper delivery vehicle, cellular uptake, immunostimulatory effect, and non-specific gene targeting and toxicity [11]. To avoid such complications, the chemical modification of siRNA structures was attempted. According to the natural structure of nucleotides, the chemical modification can be placed at the phosphate backbone, the ribose moiety, or the base [12].

After 20 years of research, the United States Food and Drug Administration (FDA) and the European Commission (EC) approved ONPATTRO (patisiran, ALN-TTR02) as the first commercial RNAi-based therapeutic for the treatment of hereditary amyloidogenic transthyretin (hATTR) amyloidosis with polyneuropathy in adults in 2018 [13]. Recently, Givosiran, an δ-aminolevulinic acid synthase 1 (ALAS1)-directed siRNA-based drug, obtained approval for the treatment of acute hepatic porphyria (AHP) [14]. Table 1 lists the FDA-approved siRNA-based drugs.

## 2. RNAi-Based Gene Silencing Approaches

Small interfering RNAs (siRNAs) are long double-standard RNA (dsRNA) molecules with a 3′ overhang [21]. Small interfering RNAs are generated by ribonuclease dicer, which is an RNase III family protein, through endonucleolytic processing. RNA-induced silencing complex (RISC) is used to denote a family of heterogenous molecular complexes which can target any gene for silencing [22]. The loaded siRNA serves as the template for it to identify a complementary RNA sequence present in the target gene. Once located and identified, an Argonaute-like endonuclease present in the complex cleaves the siRNA from the complex, allowing it to bind to its target RNA to initiate silencing [23,24]. Designing siRNA is a crucial step in RNA interference (RNAi) experiments, as it influences the efficiency and specificity of gene silencing. A study by Ui-Tei et al. describes simple guidelines for selecting selective siRNA sequences for mammalian cells [25]. The siRNAs that satisfy the following four conditions can induce highly effective gene silencing: (i) A/U at the 3′ end (corresponding to the 5′ end of the antisense strand of siRNA). (ii) G/C at the 5′ end of the target sequence. (iii) Contains at least five A/Us at its 3′-terminus. (iv) The absence of GC stretches longer than nine nt. Computational tools are pivotal in refining siRNA sequences, offering a nuanced approach to enhance the specificity and efficacy. Table 2 shows a list of different in silico siRNA-designing computational tools. The in silico design of siRNAs makes it possible to predict potential siRNA candidates with reduced off-target effects and target specificity. It is essential to consider various factors when designing potential siRNAs, including conserved genome sequences, off-target effects, siRNA folding, thermodynamic properties, target accessibility, stability, and immune stimulation. The basic rules and guidelines for the design of siRNAs have been published in many reports [26,27,28].

## 3. Chikungunya Virus (CHIKV) and Severe Acute Respiratory Syndrome Coronavirus 2 (SARS-CoV-2)

Chikungunya virus (CHIKV) is a positive-sense RNA virus classified in the *Togaviridae* family. CHIKV infection leads to Chikungunya fever, characterized by a sudden onset of symptoms, including fever, severe joint pain, muscle pain, headache, nausea, fatigue, and rash. Although the majority of people make a full recovery, some individuals may experience enduring joint pain and symptoms similar to arthritis [29]. CHIKV is primarily transmitted to humans by infected *Aedes* mosquitoes, including *Aedes aegypti* and *Aedes albopictus* [30]. *Aedes* mosquitoes acquire the virus by feeding on viremic human hosts, allowing the virus to replicate within the mosquito before being transmitted to new hosts during subsequent blood meals. Vertical transmission from an infected mother to her newborn and, in rare cases, through blood transfusions have been reported [31]. CHIKV contains a linear, positive-sense, single-stranded RNA genome of approximately 11.8 kb length. The genome consists of a capped 5′ non-coding region (NCR) and 3′ polyadenylated NCR. The non-structural proteins nsP1, nsP2, and nsP3 are important proteins for virus replication; the structural proteins, which consist of capsid and envelope proteins (E1, E2, E3, and 6K), are synthesized as polyproteins and are cleaved by capsid autoproteinase and signalases [32].

SARS-CoV-2 is a novel coronavirus, part of the *Betacoronavirus* genus within the *Coronaviridae* family [33]. The virus is responsible for the global COVID-19 pandemic [34]. The symptoms of COVID-19 include fever, cough, shortness of breath, fatigue, loss of taste or smell, and in severe cases, pneumonia and acute respiratory distress syndrome (ARDS). SARS-CoV-2 primarily spreads through respiratory droplets from infected individuals produced during coughing, sneezing, or talking [33]. The key proteins of SARS-CoV-2 include the spike (S) protein, envelope (E) protein, membrane (M) protein, nucleocapsid (N) protein, and RNA-Dependent RNA Polymerase (RdRP) [35].

### 3.1. RNAi against CHIKV Infection

The first in vitro study by Das et al. [36] showed the effectiveness of siRNAs against non-structural protein 3 (nsP3) and envelope protein 1 (E1) for the inhibition of Chikungunya virus (CHIKV) replication in Vero cells. This study depicted a 99.6% reduction in CHIKV titers in siRNA-transfected cells 24 h post infection, but the reduction was not sustainable until 72 h after infection due to the possible intracellular degradation of siRNA [36].

In 2012, we designed the very first siRNAs against the non-structural protein 1 (nsP1) and envelope 2 (E2) proteins of CHIKV and evaluated their antiviral activity under both in vitro and in vivo conditions [37]. Our data clearly confirmed that the combination of siRNAs could completely inhibit CHIKV replication by simultaneous silencing of multiple genes and without eliciting any unwanted adverse reactions during in vivo studies. The proper delivery of siRNA formulation is another essential approach for successful siRNA-based treatment. To further strengthen our findings, we conducted multiple studies to find an effective siRNA delivery mechanism for inhibiting CHIKV infections. Until now, most studies have used lipid nanoparticles, polymeric nanoparticles, gold nanoparticles, iron oxide nanoparticles, mesoporous silica nanoparticles, and porous silicon nanoparticles for the efficient delivery of siRNAs. Recently, we reported that a novel cationic lipid nanoparticle-loaded siRNA with stearylamine can reduce CHIKV infection. In this study, a nanodelivery system using cationic lipids and polymers was synthesized and complexed with siRNA. The data suggested that a delivery system containing stearylamine with a charge of 45.7 mV allowed better stability, complexation, stable transfection, and potential inhibition of the E2 and NS1 genes of CHIKV [38]. Metal–organic frameworks (MOFs), a class of porous, highly ordered crystalline polymers, have been trialed as delivery vehicles for drugs. Zeolitic imidazolate frameworks (ZIFs), a subtype of metal–organic frameworks, are safe and efficient delivery vehicles for gene editing components. Their advantageous characteristics, including excellent biocompatibility, adjustable pore size, and a rigid molecular structure, make them ideal candidates for such applications [39] A recent study conducted by us utilized a ZIF-8 analog, ZIF-C, as the delivery system for siRNA in CHIKV-infected cells. ZIF-C biocomposites were used to deliver the siRNAs into CHIKV-infected Vero cells which then initiated targeted silencing of the E2 and nsP1 genes of the Chikungunya virus in the infected cells, effectively curbing viral replication and pathogenesis in vitro [40]. The successful in vitro delivery of siRNA through this biocomposite warranted further studies involving in vivo models.

### 3.2. RNAi Approaches for the Treatment of SARS-CoV-2 Infections

During the COVID-19 pandemic in late 2019, there was a significant increase in siRNA research to find an alternative solution. The first siRNAs against SARS-CoV-2 were designed by Chowdhury et al. [41]. The group designed eight potential siRNAs using computational methods based on conserved sequences in the nucleocapsid phosphoprotein and surface spike glycoprotein gene utilizing a dataset of 139 strains [41]. Later on, another study identified nine different siRNA target sequences using a single reference sequence through computational study [42]. The designed siRNAs were located in Orf1ab, Orf1b, S gene, Orf3a, the M gene, and the N gene. Later on, our group also predicted potential siRNA molecules using a larger dataset of ~6000 different SARS-CoV-2 sequences [43]. This study followed the standard screening protocol and different parameters to increase the probability of designing highly functional siRNAs against SARS-CoV-2. Three of these siRNAs were targeted toward the HR1 nucleotide region of the S gene. Another study by Madanagopal et al. [44] also designed and predicted four siRNA molecules against the envelope gene (E), membrane gene (M), nucleocapsid phosphoprotein gene (N), and surface glycoprotein gene (S) using 811 Indian strains of SARS-CoV-2 [44]. None of these studies evaluated the in vitro or in vivo efficiency of the designed siRNAs. Later, Tolksdorf et al. [45] designed siRNAs against the 5′-UTR of the SARS-CoV-2 genome by selecting a crucial conserved region for viral RNA replication and transcription and performed the experiments in a Vero E6 cell line [45]. The group confirmed that a set of eight siRNAs can efficiently target the 5′-UTR of SARS-CoV-2 and can inhibit viral replication. The most efficient siRNA, siCoV6, binds to the highly conserved transcription regulatory sequence (TRS) and targets the leader sequence. Similarly, in 2022, Sohrab SS et al. screened three siRNAs out of twenty-six siRNAs using in silico and in vitro assessment of an HEK-293 cell line [46]. Another study by Rohani et al. in 2021 used computational and bioinformatics approaches to narrow down the cases for experimental validation. Their study reported five efficient siRNAs targeting four crucial regions in SARS-CoV-2 including 0RF1ab, nsp3; ORF1ab, nsp13; ORF1ab, nsp4; and spike protein [47].

## 4. Treatment with Immunomodulatory and Antiviral Oligonucleotides: A Promising Dynamic Duo

The host immune response plays a significant role in the pathogenesis of many viral diseases. Drugs targeting the host factors as well as the virus might be more effective compared to those that aim at only one component [48]. The endosomal compartments of the cell express certain Toll-like receptors (TLRs) that are capable of recognizing foreign nucleic acids or microbes. A key function of these receptors is to trigger the body’s response to infections caused by viruses and bacteria [49]. The identification of viral RNA and the initiation of the immune response against viral infections both depend on the TLR3 receptor. Hyperactive reactions, however, might have negative consequences, including virus-induced asthma and other illnesses. Numerous illnesses, including viral infections, have been linked to excessively reactive TLR3 signaling triggered by viral or endogenous dsRNA from dying cells. It is possible that siRNAs might also be recognized by the immune system, leading to an inflammatory response. Recent studies discovered that TLR3 activation was inhibited by a set of ssDNA that acts in a sequence-independent manner. TLR3 activation can be inhibited by this class of single-stranded oligonucleotides (ssONs) by temporarily blocking clathrin-mediated endocytosis, preventing the uptake of the TLR3 ligand dsRNA [50,51,52]. For therapeutic oligonucleotide-based therapy, it is preferred to evade immune recognition to achieve maximal pay-off. Targeting virus entry is one of the possible mechanisms of an antiviral drug because it interrupts the viral life cycle and prevents subsequent replication and pathogenic processes. As a result, it was prudent to investigate whether ssONs capable of inhibiting endocytosis could be used as an antiviral agents [53]. Through further experiments, it was found that ssONs between 25 and 35nt could inhibit RSV infection, and the said property was dependent more on its length than its sequence or origin (RNA or DNA) [53,54]. Thus, on one hand, it serves the purpose of a broad-spectrum antiviral and on the other, it prevents any adverse immune reaction to the administered drug by impeding TLR3 activation. This culminates in a possible excellent delivery system combined with efficacy. Therefore, the usage of immunomodulatory and antiviral oligonucleotides in combination with siRNA may provide a legitimate and effective strategy to deal with viral infections.

## 5. Current Status of siRNA-Based Therapeutics for Other Human Viral Diseases

**Figure 1:**
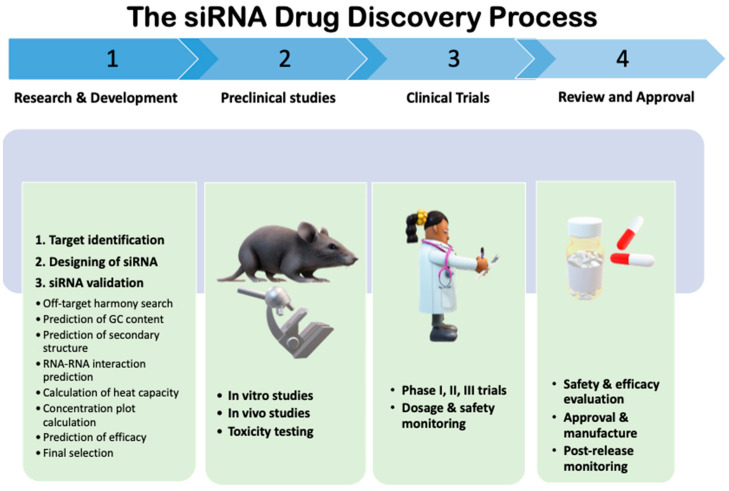
The siRNA drug discovery process.

More focused work on siRNA-based therapeutics might yield an effective solution for various diseases. According to the FDA, there are three main phases of clinical trials—phases I to III—and some phase IV trials are also performed when the drug becomes licensed (Figure 1) [55]. During phase I, the formulated drug is tested on a small number of volunteers to evaluate its safety, doses, and side effects. During phase II, a large group of patients are monitored to identify side effects, whereas continuous monitoring of a large group of patients is generally performed in Phase III.

Few research groups have succeeded in proceeding to the clinical trial stage, whereas few others were unable to progress to the phase II trials. After the approval of patisiran, givosiran, and lumasiran by the FDA, no phase IV trial data have been published for siRNA drugs. Currently, seven siRNAs are undergoing phase III clinical trials [56]. At present, 48% of clinical studies accessing siRNA candidates are already completed, whereas 40% are still active. In terms of antiviral siRNAs, ALN-RSV01 was the first RNAi therapy approved for clinical trials, which targeted the mRNA of respiratory syncytial virus (RSV) nucleocapsid protein [57]. siRNAs against hepatitis B and Ebola virus infection have also been used in clinical trials and showed excellent antiviral efficacy in infected patients [58,59]. RNAi gained massive popularity due to its fastest gene knockdown mechanism. Table 3 provides the current status of various antiviral siRNAs.

## 6. Barriers Affecting the Use of siRNA for Therapeutic Purposes and Possible Solutions

The advancement of the latest bioinformatics-based analysis methodologies and easy development protocol have generated great hope in this field. However, applying siRNA-based therapeutics in humans is difficult due to several factors, most importantly the lack of a perfect delivery mechanism.

The most prominent hurdle is the route of administration, which inevitably gives rise to the problem of siRNA stability. As siRNA is unstable in the intestinal environment and has a reduced permeability through epithelial cells, it cannot be administered orally. On the other hand, the subcutaneous route is limited by lipophilicity and carrier size. The most effective route for the delivery of siRNA is intravenous administration; however, a suitable delivery system is required to avoid degradation or aggregation of the naked siRNA [65,66,67]. In the serum, naked siRNA is degraded by several serum endonucleases and ultimately cleared by the kidneys, resulting in a half-life less than 10 min long. The naked siRNA is also coated with proteins, making it more likely that reticuloendothelial macrophages will phagocytose it. Problems arise from liposomal carriers, where their positive charge results in aggregation along with red blood cells [67]. Newly developed nano-carriers and RNA backbone modifications have been introduced to overcome this barrier. Recently, Supramaniam et al. (2023) developed an intranasal (IN) siRNA encapsulated lipid nano particle in vivo delivery system to treat SARS-CoV-2 and respiratory syncytial virus (RSV)-mediated lung infection [68].

The intracellular barriers include endosomal trapping, traveling to the correct site, and off-target effects. siRNA molecules can bind to any mRNA that shares a sequence with the target mRNA, leading to off-target effects and the unintended silencing of other genes. siRNA therapy could lead to non-specific gene targeting. This can result in potential side effects or other unintended consequences. There are two general mechanisms behind off-target siRNA effects: First, siRNAs can tolerate multiple mismatches on the target mRNA, forming incompletely complementary pairs that continue to suppress the target mRNA. siRNA sense strands were complementary to these transcripts’ 3′ UTRs. A second reason is that external siRNAs will compete with endogenous miRNAs because the RNAi level in the body is saturated when they enter the cells. As a result, common proteins like RISC are knocked off [69,70]. Studies have shown that specific structures of siRNA sequences and drug carriers can induce the release of cytokines such as IFN-α and IFN-γ. Therefore, siRNA drugs can trigger innate immune responses [67].

Another main limitation is that siRNA-based drugs are primarily delivered to liver hepatocytes, which limits their application to diseases that primarily affect the liver. This is because the liver is the primary site for the degradation of siRNA molecules and is also the site of the highest expression of the enzyme responsible for the uptake of siRNA. It may be necessary to make a ‘second siRNA drug breakthrough’ concerning other extra-hepatic siRNA delivery platforms and applications in addition to advances in the central nervous system and ocular and renal siRNA delivery.

The appearance of mutations in the siRNA-binding region might challenge the development of siRNA-based therapeutics for viral infections. Targeting conserved regions in the genome might help to combat viral infections. Researchers have used various approaches to overcome such hurdles. Several studies introduced chemical modifications in the siRNA, which helped in both stability and immune escape [71,72]. The use of various ligand–nanoparticle conjugates helps avoid non-specific binding and kidney filtration [73]. Encapsulating siRNA into vesicles and conjugating it with ligands increase on-site target binding [74]. Many studies introduced cationic cell-penetrating peptides (CPPs), which helps the cellular uptake of siRNA by introducing pore formation. All the siRNA drugs that have undergone clinical trials used various delivery systems, including lipid nanoparticles (LNPs), Dynamic PolyConjugate (DPC^TM^), Targeted RNAi Molecule (TRiM^TM^), N-acetylgalactosamine (GalNAc)-siRNA conjugates, LOcal Drug EluteR (LODER^TM^) (expansions) polymers, exosomes, and polypeptide nanoparticles [75,76,77]. Developing more stable siRNAs, achieved through advanced chemical modifications, has enabled direct conjugation with cell-specific ligands. One of the most successful approaches is the conjugation of siRNAs to N-acetylgalactosamine (GalNAc), a sugar molecule that binds specifically to asialoglycoprotein receptors (ASGPRs) on hepatocytes [77]. ASGPRs are highly expressed on the surface of liver cells, and their rapid recycling allows for the efficient uptake of GalNAc-conjugated siRNAs with a single administration [78]. GalNAc conjugation’s ability to deliver siRNAs efficiently and selectively to hepatocytes has resulted in higher clinical rates than earlier delivery methods, such as LNPs. This approach not only improves the pharmacokinetic and pharmacodynamic properties of siRNA therapies but also reduces the frequency of administration, enhancing patient compliance.

## 7. Discussion

RNA interference (RNAi) has emerged as a highly adaptable and versatile tool since its discovery, allowing researchers to modify it for various applications such as reverse genetics, specific gene repression, and targeted therapy development. The components of the RNAi pathway are not fixed but instead are constantly evolving, enabling gene-specific regulation. This adaptability is evident in new technologies that leverage RNAi foundations to enhance synthesis, functionality, potency, stability, and pharmacology, as well as to develop novel delivery methods and therapeutic strategies. Recent FDA approvals, such as inclisiran [18], the fourth therapeutic siRNA, and the progress of numerous RNAi-based therapeutics in clinical trials underscore the growing important of RNAi in medicine. siRNA molecules hold immense potential in revolutionizing drug development processes due to their ability to rapidly design and synthesize therapeutic agents. This technology offers a promising approach to achieving therapeutic effects with lower doses and fewer side effects compared to traditional drugs. Furthermore, siRNA therapies can potentially target novel disease targets that are challenging to address with conventional drugs, particularly RNA viruses. The rapid evolution of RNA viruses, including CHIKV and SARS-CoV-2, poses a challenge for RNAi therapy, as viral mutations could potentially lead to the emergence of resistant strains. The continuous monitoring of viral genetic diversity and the development of novel RNAi strategies targeting conserved regions of the viral genome will be crucial to mitigate this risk.

Despite these advantages, the widespread adoption of siRNA-based therapies faces significant challenges, primarily related to delivery. Current delivery methods often lack efficiency and specificity, necessitating the exploration of novel delivery approaches. Systems such as solid lipid nanoparticles, metal–organic frameworks, and others hold promise but require further investigation to enhance their efficacy and safety profiles. Another critical consideration for the future of siRNA-based drugs is the requirement for storage temperatures between −20 °C and −80 °C. This poses a significant challenge in resource-limited settings, where maintaining such low temperatures may be impractical. Research efforts aimed at increasing the stability of siRNA at higher temperatures, such as 4 °C to 8 °C or even at ambient temperatures, are crucial for expanding the accessibility of siRNA therapies. Looking ahead, the integration of CRISPR-based technologies with siRNA therapies represents a promising avenue for enhancing the precision and efficiency of gene silencing. CRISPR technologies enable targeted gene editing, complementing the gene-silencing capabilities of siRNA. By combining these two approaches, researchers aim to achieve more specific and efficient silencing of disease-causing genes, paving the way for personalized medicine approaches.

The success of mRNA vaccines, particularly during the COVID-19 pandemic, has highlighted the potential of lipid nanoparticles (LNPs) and other delivery technologies in nucleic acid-based therapies. These formulations have efficiently protected mRNA from degradation and ensured its delivery to target cells. Similarly, siRNA therapeutics require robust delivery mechanisms to ensure stability and effective cellular uptake. Leveraging the established mRNA vaccine delivery technologies, such as LNPs, could accelerate the development of siRNA-based therapies. However, there are challenges, including differences in dosing requirements, duration of action, and potential immune responses. Understanding these nuances is crucial for effectively adapting mRNA delivery systems to siRNA therapeutics.

While most RNAi therapeutics currently in use are siRNAs, the field of microRNA (miRNA)-based therapies also holds great promise for the future. miRNAs have a unique ability to target multiple mRNA transcripts simultaneously, offering a broader scope for therapeutic interventions. Despite this potential, few miRNA-based therapies have advanced to clinical trials, indicating an untapped opportunity for their application in medicine. Combining siRNA, which targets the virus, with miRNA, which targets the host factors that aid in virus replication and host immune response, can be a novel therapeutic approach to overcome the problem of emergence of resistance and needs further exploration. Addressing these challenges will be crucial for realizing the full potential of RNAi-based therapies. Continued research and innovation in delivery systems, stability enhancement, and storage conditions are essential.

## Figures and Tables

**Table 1:** FDA-approved siRNA-based drugs.

Drug Name	Disease	siRNA Carrier	Target Gene	Reference
Patisiran (Onpattro)	Hereditary transthyretin-mediated amyloidosis (hATTR amyloidosis)	Lipid nanoparticles	Transthyretin (TTR)	[15]
Givosiran (Givlaari)	Acute hepatic porphyria (AHP)	GalNac conjugation	Aminolevulinate synthase 1 (ALAS1)	[16]
Lumasiran (Oxlumo)	Primary hyperoxaluria type 1 (PH1)	GalNac conjugation	Hydroxy acid oxidase 1 (HAO1)	[17]
Inclisiran (Leqvio)	Primary hyperlipidemia (high cholesterol)	GalNac conjugation	Proprotein convertase subtilisin/kexin type 9 (PCSK9)	[18]
Vutrisiran (Amvuttra)	Polyneuropathy caused by hereditary transthyretin-mediated amyloidosis (hATTR amyloidosis)	GalNac conjugation	Transthyretin (TTR)	[19]
Nedosiran (Rivfloza)	Primary hyperoxaluria type 1	GalNac conjugation	Hepatic lactate dehydrogenase	[20]

**Table 2:** Software used for in silico siRNA screening (accessed on 5 December 2023).

Software Name	URL
siDirect v2.1	https://sidirect2.rnai.jp/
siRNA Scales	http://gesteland.genetics.utah.edu/siRNA_scales/
siExplorer	http://rna.chem.t.u-tokyo.ac.jp/cgi/siexplorer.htm
OligoWalk	https://rna.urmc.rochester.edu/cgi-bin/server_exe/oligowalk/oligowalk_form.cgi
Sfold	https://sfold.wadsworth.org/cgi-bin/index.pl
DSIR	http://biodev.cea.fr/DSIR/
RNAxs	rna.tbi.univie.ac.at/cgi-bin/RNAxs/RNAxs.cgi
i-Score Designer	https://www.med.nagoya-u.ac.jp/neurogenetics/i_Score/i_score.html
OptiRNA	http://optirna.unl.edu/
Ambion	https://www.hsls.pitt.edu/obrc/index.php?page=URL1069096632

**Table 3:** Antiviral siRNAs in clinical trials.

Target Virus	Drug Details	Target Gene	Delivery System	Current Status	Sponsor	References
Respiratory syncytial virus (RSV)	ALN-RSV01	Nucleocapsid gene	Naked siRNA	Phase 2, 2b, completed	Alnylam Pharmaceuticals	[60]
Hepatitis B virus	VIR-2218	X coding region	GalNAc-conjugated	Phase 2	Brii Bio	[61]
	ALN-HBV	Conserved region of HBV genome	GalNac-siRNA conjugates	Phase ½, terminated	Alnylam Pharmaceuticals	[61]
Human immunodeficiency virus (HIV-1)	LVsh5/C46 (Cal-1)	CCR5	Self-inactivating lentiviral vector based on HIV-1 backbone	Phase 1/2, completed long-term follow-up phase 1, suspended	Calimmune, Inc	[62]
	rHIV7-shl-TAR-CCR5RZ	Tat/Rev common exon	Recombinant lentivirus vector HIV7	Phase ½, recruiting	AIDS Malignancy consortium	[63]
Hepatitis C virus	TT-034	HCV Genome	Adeno-associated viral vector 8	Phase 1/2	Benitec Biopharma	NA
Zaire ebolavirus	TKM-100802	Viral polymerase L and VP35	LNPs	Phase 1, terminated	Arbutus Biopharma	[64]
	TKM-100802	Target regions of the viral polymerase L and VP35	LNPs	Phase 1, terminated	Arbutus Biopharma	

## Data Availability

Data sharing is not applicable to this article. No new data were created or analyzed in this study.
